# Vector coding reveals the underlying balance control strategies used by humans during translational perturbation

**DOI:** 10.1038/s41598-022-24731-3

**Published:** 2022-12-05

**Authors:** Naser Taleshi, James M. W. Brownjohn, Sarah E. Lamb, Stana Zivanovic, Genevieve K. R. Williams

**Affiliations:** 1 Public Health and Sports Sciences Department, University of Exeter Medical School, Exeter, EX1 2LU UK; 2 Vibration Engineering Section, College of Engineering, Mathematics and Physical Sciences, University of Exeter, Exeter, EX4 4QF UK

**Keywords:** Biomedical engineering, Civil engineering, Mechanical engineering, Anatomy, Musculoskeletal system

## Abstract

Postural control research has focused on standing balance experiments on platforms moving with relatively large amplitudes (0.1–0.2 m). This study investigated balance strategies while standing on a platform moving 4 mm in anterior-posterior direction with frequency scaled linearly from 0.4 to 6 Hz. Platform motion and kinematic and kinetic information for nine healthy participants were recorded using motion capture and force plate systems. Coordination between hip, knee and ankle joint torque, and centre of mass (COM) and centre of pressure (COP) motion was quantified by vector coding. Significant main effect of platform frequency for knee-ankle and COP-COM phase relationship was observed (p = 0.023, p = 0.016). At frequencies below 2.11 and 2.34 Hz, ankle strategy was recruited. With ankle strategy, in-phase COP-COM motion with COP dominancy occurred at frequencies below 2.19 and 2.23 Hz during scaling up and down, respectively. As platform frequency passed these values, COM dominated over COP which was followed by anti-phase knee-ankle torque, called a knee strategy, and anti-phase motion between the COP and COM that allowed COP to regain dominance over COM. Collectively, we reveal knee strategy as a new and relevant strategy in real-life settings, and transition between ankle and knee strategies that underpinned transition between COP-COM relative motion.

## Introduction

One remarkable mechanism humans display is maintaining upright balance as a bipedal species. Balance control involves the integration of visual, vestibular, and proprioceptive information^1^ which informs strategies at the level of muscle activity. External influences such as platform motion can further challenge balance control. This can be experienced daily on moving platforms e.g. floors in moving transport, swaying buildings, etc, due to changes in velocity (acceleration) that affect balance. This work aims to better understand healthy balance control strategies elicited when floor motion representing real-world situations is present.

The balance strategy used to cope with moving floors depends on different variables, including but not limited to direction, amplitude, and frequency of the perturbation^2^. Previous studies have combined various amplitudes and frequencies of a moving platform to investigate how and why our postural system changes its balance control strategy^3–6^. Ko et al.^6^ presented one of the most comprehensive studies exploring potential strategies across a range of amplitudes and frequencies of platform motion. Six frequencies (0.19, 0.38, 0.54, 0.92, 1.28, and 1.64 Hz) and three amplitudes (10, 16, and 23 cm) of the postural platform in the anterior-posterior (AP) direction were combined to examine the coordination pattern at the joint space level. They identified four distinct postural coordination modes as a function of increasing frequency: a rigid mode, ankle mode, ankle-hip mode, and ankle-hip-knee mode, respectively^6^. Underpinned by the dynamical systems theory perspective, a number of nonequilibrium phase transitions have been identified in human movement ^7^. The key to identifying these phase transitions was to capture changes in a coordination dynamic^8,9^. Thus, to provide information on the nature of the phase transition between different balance control strategies in response to the underfloor motion, the platform’s motion was suggested to be linearly scaled, while the balance control strategies were continuously quantified. For example, recent studies ^10–13^ of platform frequency at ranges of 0–3 Hz with amplitudes of 9–20 cm revealed transition between in-phase and anti-phase coordination modes in both upper levels of space (centre of pressure (COP) and centre of mass (COM) phase relationship) and the lower level of joint (lower limb joint relative motion). Dutt-Mazumder et al. ^11^ identified an in-phase-to-anti-phase change for COP-COM relative motion while the platform moved in medio lateral (ML) direction at a frequency of 0.2–1.2 Hz and a constant amplitude of 20 cm. Transition in joint space level between ankle-hip coordination was also found while platform frequency scaled within the range from 0 to 3.0 Hz at a constant amplitude of 9 cm in AP direction^10^. However this study was limited by the relatively large step size changes of frequency of 0.1 Hz. To provide more precise transition frequencies in COP-COM and ankle-hip coordination, a recent study^13^ scaled platform frequency from 0.2 to 0.8 Hz in small steps of 0.02 Hz and constant amplitude of 10 cm in the AP direction. An intriguing finding in studies on phase transition for COP-COM and ankle-hip coordination is that despite perturbing participants in different amplitudes, the transition frequency has been reported between a comparatively narrow frequency range between 0.4 Hz and 0.6 Hz, 0.4–0.5 Hz for AP^10,13^, and 0.4–0.6 Hz for ML direction ^11,12^. These studies mainly addressed postural strategies with relatively large the amplitudes of underfoot motion, in the range 9–23 cm i.e. larger than those found in day-to-day life and predominantly reported ankle and hip strategies for maintaining balance. This limits the application of the results and might also explain why the transition frequency is predominantly reported in the narrow range of 0.4–0.6 Hz. The 3D solution space provided in this study, Figs. 3 and  4, illustrates that ankle, knee, hip, and combined strategies are possible solutions to balance control offered by central nervous system (CNS) and could emerge under different environmental conditions. In this regard, it is possible that the full scope of compensatory postural strategies has not been identified yet. Our study used a unique facility called VSimulators to precisely provide AP floor movement at a small amplitude and a wide range of frequencies, never before explored, but that more closely reflect real-world floor motions^2^.We hypothesized that there might be a broader set of possible coordination modes available than heretofore observed that allow postural balance and stability on the substantially lower magnitude level than the previous studies. We also hypothesized that as the platform frequency reaches a critical value it would cause a loss of stability and induces a new search process for new stable region or adaptive compensatory coordinative mode.

Previously, the definition of the postural control strategies has been based on joint kinematics through their relative phases^6,8,9,13–15^. The phase relationship between joint or segment kinematics helps describe body movement; however, joint torques can provide insight into the mechanism of joint motion. Movement of body segments can be caused by muscle activation at that joint, external forces or by interactive torques from other segments. For instance, the hip joint can be flexed either because of hip torque or ankle plantarflexion coupled with gravitational forces acting on the trunk^16,17^. Therefore, it has been suggested that joint torques can be used to identify postural strategies^16^. Yeadon and Trewartha ^18^ distinguished different postures using correlation analysis on adjacent joint torques in the handstand posture. Blenkinsop et al.^19^ extended this model to a multi-segment planar model for standing balance and identified postural control strategies by multiple correlations of an adjacent joint comprising knee, ankle, and hip torque. As well as joint level definitions, COM and COP phase relationship has been suggested as the collective variable that describes the organization of the multi-segmental whole-body postural control system^10^. In order to follow this line of research we investigated how the postural system regulates the joint torque space degree of freedom (DOF) to govern the COP-COM coordination.

The relative phase between joint and segment kinematics has predominantly been investigated by the point estimate relative phase (PRP)^20^ and continuous relative phase (CRP) method^9^ across different platform frequency conditions. These PRP and CRP methods have revealed different dynamic phenomena accompanying a nonlinear phase transition, such as hysteresis and critical fluctuations^9^. However, PRP is limited by only capturing relative phase at a single instance per cycle, while CRP is inherently difficult to interpret^21^. Originally introduced by Sparrow et al.^22^, and further quantified by Hamill et al.^23^ and Tepavac and Field-Fote^24^, vector coding (VC) is a technique to quantify the coupling between pairs of oscillators over an entire time series that is inherently interpretable. Variations for capturing coupling have been presented by Chang^25^, Needham^26,27^ and Hamill^23^ and used in a number of different scenarios particularly in gait analysis^25–31^. To the best of the authors’ knowledge, no previous research has investigated the postural coordination modes of the joint or the COP-COM motion using the VC method during a standing balance task. With the power to quantify relative phase throughout the full range of phase relationships across the entire time series and the ability to quantify the dominancy/relative amplitude of the motion of one oscillator compared to the other^25,26^, we believe VC can be very informative in this context. For example, COP is often assumed as the controlling variable while the COM is assumed as the controlled variable^32,33^. Therefore, the dominancy of COP motion could indicate a more stable state^32^.Consequently, we may be able to identify additional coordination tendencies to the patterns presented in current literature. Therefore, the present research examined the range of stable patterns for lower limb joint torques and COP-COM coordination using the VC method^2^ while test subject is exposed to AP motion of the support surface.

## Methods

### Participants

The values reported in a similar study’s result^12^ is used to calculate the effect size^34^. Then, to detect a Cohen’s effect size of f = 0.20, with 95 percent power in a two-way ANOVA test, alpha = 0.05, a power analysis carried out with G* Power^35^ suggested that nine participants is needed for this study. Therefore, nine healthy and novice subjects (five males and four females (mean age: $26.1\pm 4.7$ years, mass: $68.4\pm 5.4$ kg; height: $1.68\pm 0.07$ m) participated in this study. Prior to data collection ethical approval was obtained from University of Exeter sport and health sciences’s research ethics committee. All participants provided written informed consent and all methods were performed in accordance with the relevant ethics guidelines and regulations. Exclusion criteria for this study were any prior experience of balance control tasks or exposures to vibration and any relevant disorder history such as balance-related problems, recurrent falls, dizziness, vision problems, or vestibular dysfunction. Participants were informed that the experiment could induce motion sickness.

### Apparatus

**Figure 1:**
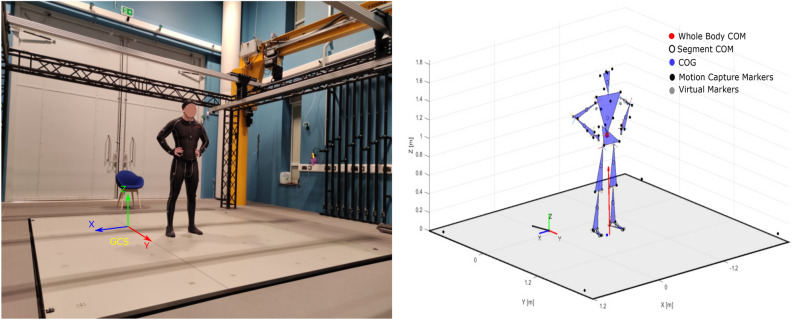
Experimental apparatus and setup. The left panel shows the moving floor embedded with force plates that oscillate sinusoidally along the AP direction, with a participant standing with bare feet, eyes open and arms on his side, and a global coordinate system (GCS). Informed consent was obtained to publish the image of participant in an online open access publication. The right panel shows a schematic stick diagram of the participant standing on the platform created using the marker and force plate information in MATLAB. This figure is used to demonstrate the GCS, local coordinate system (LCS) for each joint, ground reaction force (GRF), whole-body COM, segmental COM, COG, and COP position calculated using the force and camera data.

Participants stood on a 3.6 m by 3.6 m moveable fully instrumented floor in the Exeter VSimulators facility (Fig. 1). The data collection system consisted of 24 cameras sampling at 100Hz (OptiTrack, NaturalPoint Inc., Corvallis, OR, USA). Thirty-nine markers were attached to a suit that participants donned before starting the test. The markers were positioned according to the Plug-in Gait. The position of markers is shown in Fig. 1. Four extra markers were attached to the floor to capture the floor motion. Participants’ ground reaction force and moment were collected using the AMTI force plates system (AMTI, Advanced Mechanical Technology Inc., size 120 cm by 120 cm ). Four accelerometers embedded in the platform were also used to record the acceleration of the platform (1000 Hz).

### Procedures

Participants stood with their hands on their hips, looking at a marker at 5 m in front of them at eye level, while standing on a sinusoidally translating platform with motion in the AP direction. The platform motion consisted of an initial 7 s of quiet standing with no platform motion, followed by floor motion and another 7 s of quiet standing for total duration of 146 s. The platform frequency was scaled up in 66 s and then down with the same speed. The platform frequency was linearly scaled up from 0.4 to 6 Hz while the amplitude was kept at a constant value of 2 mm peak (Fig. 8f). Before the experiment commenced, participants took part in a familiarization test to adapt to the unstable surface and the perturbation mechanism. Participants placed their feet on two taped marks on the floor to keep their bare feet in the same starting position throughout the test.

### Data pre-processing

All the data were processed in MATLAB 2019^36^. Marker histories and force plate data were filtered with a fourth-order, 8 Hz low-pass Butterworth filter. 1000 Hz force plate and accelerometer data were downsampled to a common sample rate of 100 Hz using a spline method. Whole body COM of the participant was estimated as the weighted average of each of the segmental COM information using the 15-segment model proposed by Hanavan^37^, according to anthropometric data provided by Dempster^38^. The COP trajectories were derived from the six independent signals, 3D force and 3D moment recorded from the force plate^32^. Figure 1 shows the position of estimated location for segmental COM positions, whole-body COM, COG, and COP in the GCS. Force plate inertial components were removed from the forces and COP data using the method provided in study^39^. Also, the motion capture system recorded the motion of markers relative to the stationary GCS. Thus, platform movements were included in the marker positions requiring a correction to remove platform motion before 3D inverse dynamic analysis (IDA) could be performed using a Newton-Euler method^40^.

### Quantifying coordination; vector coding and calculation of relative phase

**Figure 2:**
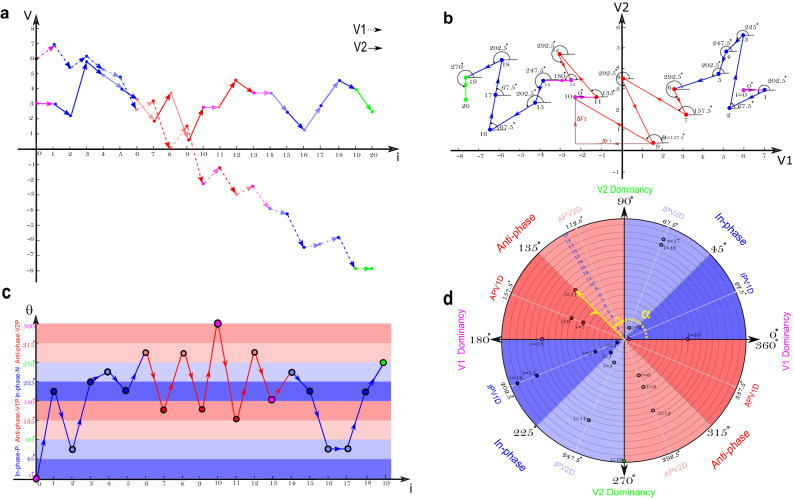
An example of extracting the coordination pattern for two synthetic variables: $$ {{V}_{1}}$$ and $$ {{V}_{2}}$$. Colour of arrows and portions emphasise the type of phase relationship between the two variables (red = anti-phase relationship, blue = in-phase relationship, pink = $$ {{V}_{1}}$$ dominancy, green = $$ {{V}_{2}}$$ dominancy. (**a**) Changes in value of synthetic variables against time. The arrows show direction of change. (**b**) State space of the variables $$ {{V}_{1}}$$ and $$ {{V}_{2}}$$. (**c**) Relative phase between $$ {{V}_{1}}$$ and $$ {{V}_{2}}$$ from instant $$i=0$$ to $$i=19$$. The phase relationships are colour-coded based on coordination pattern classification suggested by Needham et al.^26^. (**d**) Polar plots for relative phase from instant $$i=0$$ to $$i=19$$ colour-coded based on Needham et al.^26^.

To quantity the coordination between COP-COM and adjacent joint torques, we first needed to obtain their state space, illustrating the relative motion of these variables. For example, Fig. 2b presents a state space of two synthetic variables: $$ {{V}_{1}}$$ and $$ {{V}_{2}}$$ from Fig. 2a. The relative motion between the two variables was further quantified with the relative phase angle $$ \theta $$. As shown in Fig. 2b, the relative phase angle is defined between a vector connecting two consecutive data points relative to the right horizontal axis^22^. For each instant from $$ (i=0) $$ to $$ (i=19)$$ in Fig. 2b, from right-to-left, the relative phase $$ \theta $$ can be calculated based on the first and second oscillator movements, $$ {{V}_{1}}$$ and $$ {{V}_{2}}$$, according to 1:

1 $$\begin{aligned} \theta (i) ={{\tan }^{-1}}(\frac{{{V}_{2}}(i+1)-{{V}_{2}}(i)}{{{V}_{1}}(i+1)-{{V}_{1}}(i)}) \end{aligned}$$

where $$ {{0}^{\circ }}\le \theta \le {{360}^{\circ }} $$ and $$i=0,1,2,3,...19$$ represents consecutive data points. Figure 2c illustrates that the relative phase between $$ {{V}_{1}}$$ and $$ {{V}_{2}}$$, from left-to-right, transits from in-phase to anti-phase relationship at instant $$i=6$$ and from anti-phase to in-phase relationship at instant $$i=14$$. From instant $$i=1$$ to $$i=5$$ and $$i=14$$ to $$i=19$$ variables $$ {{V}_{1}}$$ and $$ {{V}_{2}}$$ create a relative phase angle between $$ {{0}^{\circ }}\le \theta \le {{90}^{\circ }}$$ or $$ {{180}^{\circ }}\le \theta \le {{270}^{\circ }} $$ which are classified as an in-phase relationship^26^. The in-phase relationship indicates both variables move in the same positive or negative direction, the solid and dashed blue arrows shown in Fig. 2a. From instant $$i=6$$ to $$i=13$$ variables $${{V}_{1}}$$ and $$ {{V}_{2}}$$ create a relative phase between $$ {{90}^{\circ }}\le \theta \le {{180}^{\circ }}$$ or $$ {{270}^{\circ }}\le \theta \le {{360}^{\circ }} $$ which are classified as an anti-phase relationship^26^. The solid and dashed red arrows in Fig. 2a illustrates that the variables move in the opposite direction which indicates their anti-phase relationship. This colour-coding method suggested by Needham et al.^26^ might help us to distinguish different phase relationship behaviour; however, representing the circular relative phase information with this linear graph might be confusing for some instants with the similar dynamic behaviours (Fig. 2c). For example, due to directional nature of relative phase, the phase relationship for both instant *i* = 0 on the down and instant $$i=10$$ on the top of horizontal bars in Fig. 2c show that there is first oscillator motion, $$ {{V}_{1}}$$ , but no second oscillator motion, $$ {{V}_{2}}$$. Thus, they both represent a similar dynamic behaviour called the first oscillator dominance phase^26^. Due to this, following changes in phase relationship for highly fluctuating data such as human kinematics and kinetics can be challenging.

A polar plot was employed to visualize the phase relationships between two oscillators as they evolve over time (Fig. 2d). In this two-dimensional coordinate system, the distance from the reference point in the centre *r* determines the progress or instant in time. The angle $$\alpha $$ represents the phase relationship between the two oscillators at that instant in time. For example, the angle $${\alpha =\theta }_{11}$$ in negative diagonal in Fig. 2d, determines the phase relationship between $$ {{V}_{1}}$$ and $$ {{V}_{2}}$$ at the instant $$ i=11 $$. The phase relationship between two oscillators can vary from $${{0}^{\circ }}$$ and $${{360}^{\circ }}$$. The polar plot for relative phase data is divided into eight portions, and colour-coded based on the coordination classification suggested in^26^. Based on the polar position of each portion, the relative phase data was categorized into four unique coordination patterns: (1) anti-phase with first oscillator dominancy, (2) in-phase with first oscillator dominancy, (3) anti-phase with second oscillator dominancy, (2) in-phase with second oscillator dominancy^25^. Figure 2d shows the four coordination patterns where the light and dark blue portions shows in-phase with $$ {{V}_{1}}$$ and $$ {{V}_{2}}$$ dominancy, respectively. The light and dark red portions represent anti-phase with $$ {{V}_{1}}$$ and $$ {{V}_{2}}$$ dominancy, respectively. An oscillator dominancy corresponds to a greater range of movement at each given moment. When phase relationship angles parallel the horizontal ($${{0}^{\circ }}$$ or $${{180}^{\circ }}$$), there is $$ {{V}_{1}}$$ motion, but no $$ {{V}_{2}}$$ meaning a $$ {{V}_{1}}$$ dominancy. Vertically directed phase relationship angles ($${{90}^{\circ }}$$ or $${{270}^{\circ }}$$) indicate a $$ {{V}_{2}}$$ dominancy , in which the $${{V}_{2}}$$ moves exclusively.

### The solution space

Blenkinsop et al.^19^ determined balance control strategies by multiple correlations of adjacent joint torques using a 4-segment model created from ankle, knee, and hip joints. A positive correlation between all the adjacent joint torques identified an ankle strategy, and a negative correlation between ankle and knee torques or knee and hip torques identified a knee or hip strategy, respectively^19^. In our work control strategies were determined by analyzing phase relationships between adjacent joint torques, rather than correlations.

**Figure 3:**
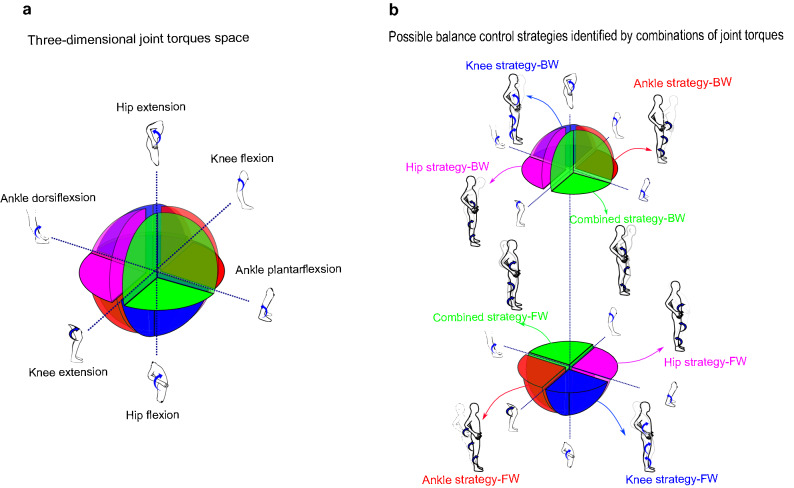
Solution space and possible balance control strategies identified by different combination of joint torque (**a**) solution space: where each axis represents one of the joint torques with anterior torques being positive. (**b**) The possible balance control strategies created from different combination of joint torques are represented with different colours.

**Figure 4:**
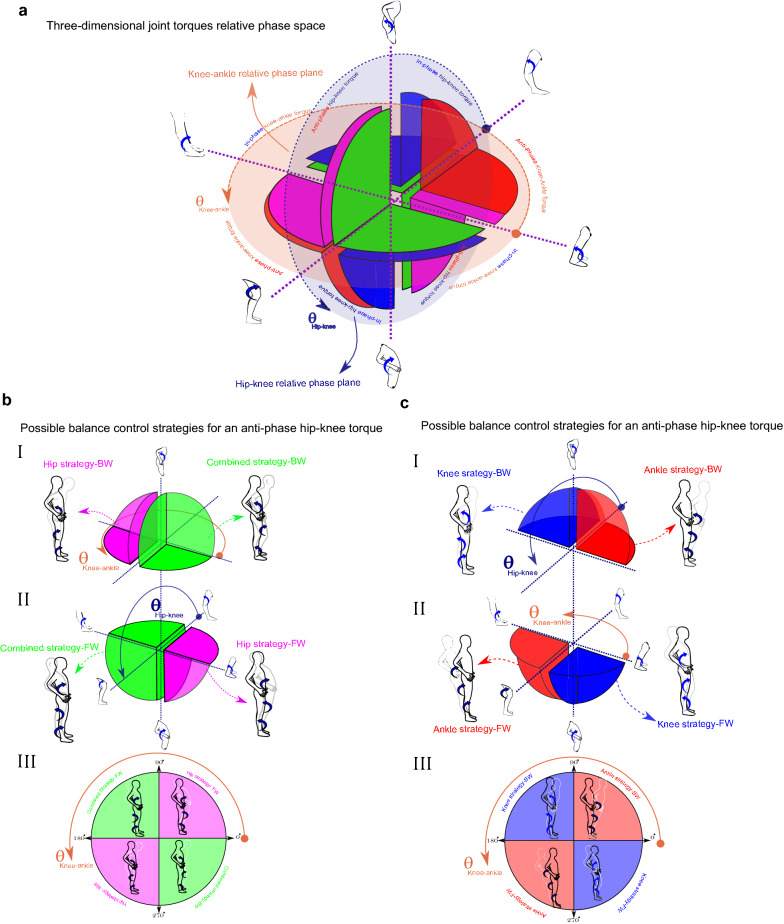
Identifying balance control strategies using hip-knee and knee-ankle torque phase relationship (**a**) solution space created from adjacent joint torque relative phases. The blue and brown plane represent hip-knee and knee-ankle joint torque relative phase. The blue and brown arrows around these planes show the change direction in the joint relative phase angle. (**b-I**, **II**) Anti-phase relationship between hip-knee joint torque resulted in either hip or combined strategy in forward (FW) or backward (BW) direction, (**b-III**) knee-ankle relative phase identifies the unique strategy used for the anti-phase relationship between hip-knee joint torque (**c**). Similarly, the in-phase relationship between hip and knee determines whether ankle or knee strategy is used. Then, a knee-ankle torque phase relationship identifies the unique balance control strategy used.

In the present study, joint torques are calculated in GCS and their phase relation are investigated in sagittal plane such that anti-clockwise torques were considered positive and clockwise torques were considered negative. Thus, positive torque signified hip extension, knee flexion or ankle plantarflexion which moved the body in the posterior direction. In contrast, a negative torque represented hip flexion, knee extension or an ankle dorsiflexion which moved body in the anterior direction. Therefore, a complete description of potential balance control strategies was accomplished with eight different combinations of the joint torques, two directions and three joints ($2^3$). If we suppose each axis of a coordination system represents one of the joint torques, the combinations of joint torques would create a 3D space called ‘solution space’^41^. The 3D solution space is shown schematically in Fig. 3a. This solution space is divided into eight divisions or octants which determine four different postural strategies (ankle strategy (red octants), knee strategy (blue octants), hip strategy (pink octants), and combined strategy (green octants) in two directions of body movements (anterior or posterior) (Fig. 3b). The red octants illustrate that synergistic torques around the knee and hip applied in the same direction with ankle torque would identify an ankle strategy. Accordingly, the hip strategy divisions (pink octants) would involve a hip torque operating in the opposite direction to both the knee and ankle torques. Similarly, the rest of the postural strategies can also be identified by analyzing the direction of joint torques^16,41^. Determining potential postural strategies can be facilitated via the analysis of phase relationship between only two adjacent joint torques^19^. In this view, a simultaneous in-phase (anti-phase) relationship between hip-knee and knee-ankle torque determines an ankle (combined) strategy. Similarly, an anti-phase (in-phase) relationship between the hip-knee torque in the presence of an in-phase (anti-phase) knee-ankle torque determines hip (knee) strategy. Therefore, investigating relative phases for only two adjacent joint torques (hip-knee and knee-ankle plane) would be adequate to distinguish the balance control strategies related to the eight octants (Fig. 4).

Figure 2d illustrates that the phase angles of ${{0}^{\circ }}\le \theta \le {{90}^{\circ }}$ and ${{180}^{\circ }}\le \theta \le {{270}^{\circ }}$ (quadrant I or III) are classified as an in-phase whereas phase angles of ${{90}^{\circ }}\le \theta \le {{180}^{\circ }}$ and ${{270}^{\circ }}\le \theta \le {{360}^{\circ }}$ (quadrant II or IV) determine an anti-phase relationship between two variables^26^. Accordingly, the anti-phase relationship for hip-knee joint torque indicates that the ${{\theta }_{hip-knee}}$ should be either in quadrant II or IV of hip-knee joint torque plane, blue plane in Fig. 4a. Therefore, the anti-phase relationship for hip-knee joint torque would limit the 3D solution space into pink or green octants, which determines whether the hip or combined strategy is used, respectively, figures I and II of Fig. 4b. Now, the knee-ankle joint torque plane, the brown plane in Fig. 4a, can identify which of the potential strategies, hip or combined, is used exclusively. A ${{\theta }_{ knee-ankle }}$ either in quadrant I or III of the knee-ankle joint torque plane in figure III of Fig. 4b shows an in-phase relationship for knee-ankle joint torque and identifies a combined strategy. A knee-ankle torque phase angle in quadrant II or IV illustrates an anti-phase relationship between them and identifies a hip strategy. Similarly, an in-phase relationship in the hip-knee joint torque plane (Fig. 4a), would limit the 3D space to only blue and red octants, figures I and II of Fig. 4c, representing the knee and ankle strategy, respectively. Then, the in-phase (anti-phase) relationship between knee-ankle joint torque indicates that the knee (ankle) strategy is used, figure III of Fig. 4c. Therefore, the polar position of relative phase data for knee-ankle and hip-knee joint torque can determine the outcome balance control strategies used.

### Polar plot and quantifying transition frequency

**Figure 5:**
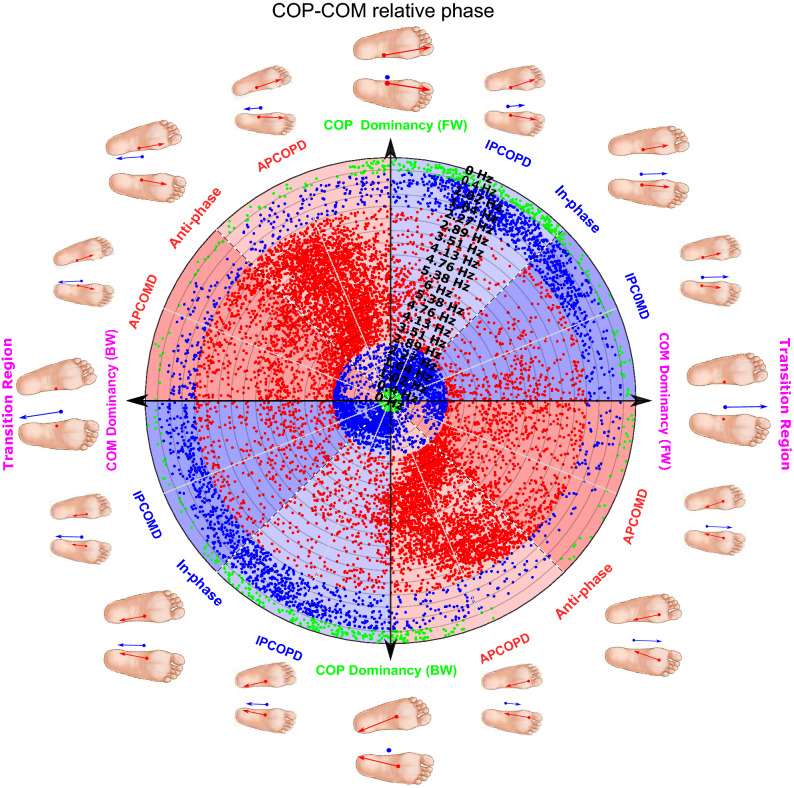
Polar plot for COP-COM phase relationship for a representative participant when the platform frequency is scaled up from 0.4 to 6 Hz over 66 s and then back to 0.4 Hz over the same period of time. The polar plot is colour-coded by coordination pattern classification suggested by Needham et al.^26^. The red and blue-colour data points signify the anti-phase and in-phase relationship between COP-COM, respectively. The green-colour data points signify the information related to the quiet standing condition. The distance from the reference point in the centre determines the progress in time or change in the platform frequency from low frequency to 6 Hz and back to low frequency. The possible coordination modes between COP and COM motion are shown around the circumference of the polar plot where IPCOPD and IPCOMD represent in-phase relationship with COP and COM dominancy, with light blue and dark blue portions respectively. APCOPD and APCOMD represent anti-phase relationships with COP and COM dominancy, with light red and dark red portions respectively. BW and FW represent backward and forward directions of body movement, respectively. The black-and-yellow and multicolour circles around the circumference of the polar plot represent COM and COP relative motion in the AP direction, respectively. The direction of COP and COM motion is signified by the direction of blue and red arrows, respectively, or a change from fade to the original colour of COP and COM circles.

**Figure 6:**
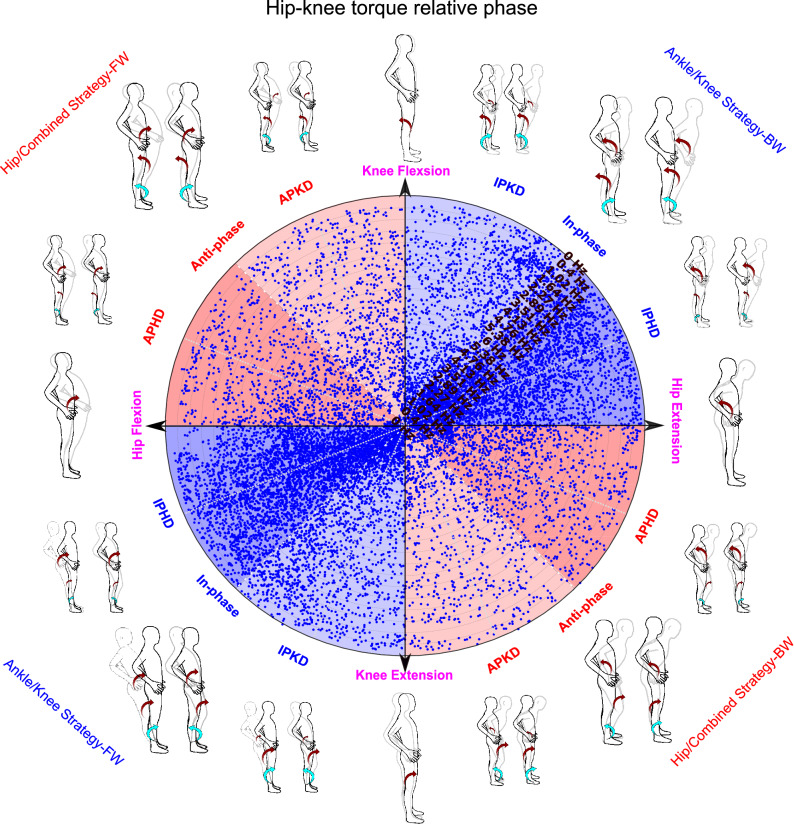
Polar plot colour-coded^26^ shows the phase relationship between hip-knee torque for the representative participant when the platform frequency scaled up from 0.4 to 6 Hz and then scaled back to 0.4 Hz again. Possible coordination patterns between hip-knee torque and corresponding balance control strategies shown around the circumference of the polar plot (IPKD (in-phase relationship with knee joint torque dominancy), IPHD (in-phase relationship with hip joint torque dominancy), APKD (anti-phase relationship with knee joint torque dominancy), APHD (anti-phase relationship with hip joint torque dominancy), BW (backward direction of body movement), FW (forward direction)). Each combination of joint torques (balance control strategies) would tend to straighten the lower limb from the semi-flexed position shown, gray-colour poses, to the black-colour straight pose). The arrows around the joints signify the required in-phase and anti-phase relationship between joint torques to create a specific balance control strategy. The direction and size of red colour arrows (related to hip and knee joint torque) are identified by their polar position in this figure. Two possible strategies can be identified for each position in the polar plot depending on the direction of the ankle joint torque (cyan-colour arrows). The blue-colour data points show an in-phase relationship between hip-knee torque and identify either ankle or knee strategy used by participants, signified by the poses around the circumference of the polar plot.

**Figure 7:**
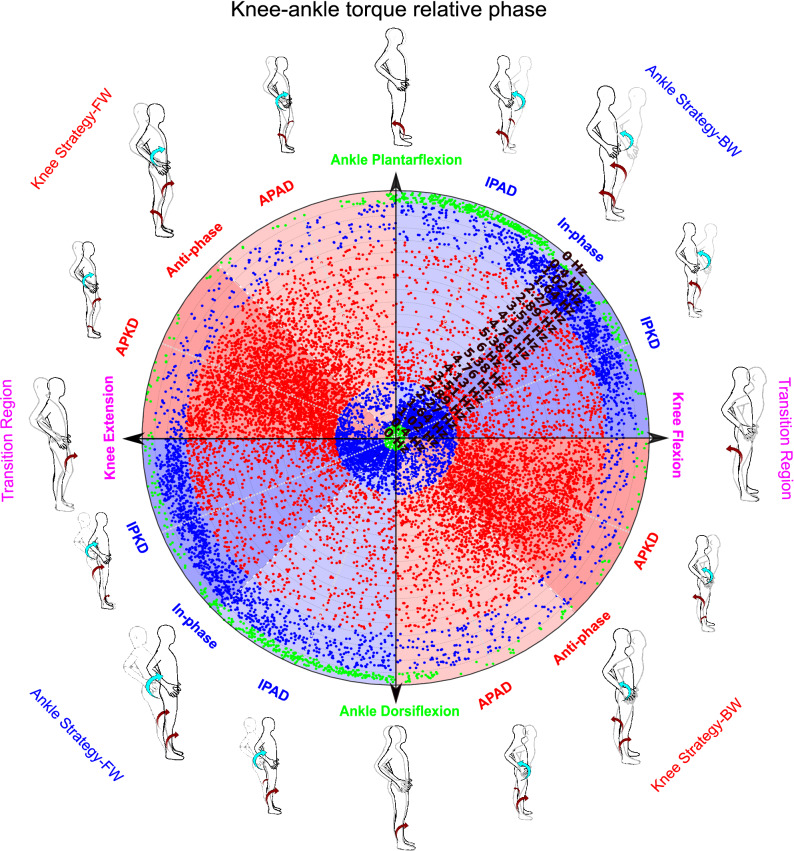
Polar plot colour-coded^26^ shows the phase relationship between knee-ankle torques for the representative participant. The possible balance control strategies are shown around the circumference of the polar plot where IPKD and IPAD represent in-phase relationship with knee and ankle joint torque dominancy, respectively. APKD and APAD represent anti-phase relationship with knee and ankle joint torque dominancy, respectively. The arrows signify the required in-phase and anti-phase relationship between joint torques to create a specific balance control strategy. The direction and size of red-colour arrows (related to ankle and knee joint torque) are identified by their polar position in this figure. According to Fig. 6, the hip-knee joint torque remained in-phase throughout; therefore, hip joint torque (cyan-colour arrows) is depicted in-phase with the knee all the time. The red and blue data points signify the anti-phase and in-phase relationship between knee-ankle joint torques and identify knee and ankle strategies, respectively. The green data points signify the data related to the quiet standing condition.

The polar plots for relative phase between adjacent joints torque (Figs. 6, 7), and COP-COM displacement (Fig. 5), visualize the transition region in synergy and space level of Bernstein model^42^, respectively. The polar plot for hip-knee and knee-ankle torques shows how the joint torques change correspondingly over time (Figs. 6, 7). Similarly, the polar plot in Fig. 5 shows how COP and COM move accordingly. The horizontal and vertical coordinates of the polar plots for the relative phase angle determine the proportion and direction of each oscillator at each instant in time. For example, a positive (negative) displacement of COM and COP signified anterior (posterior) movements. The platform frequency was continuously scaled up first and then scaled down; therefore, each data point in these plots represents a phase relationship between two adjacent joint torques or COP-COM motion at a specific frequency. However, only 21 levels related to 21 increasing and decreasing frequency levels were titled in the polar plots. As shown earlier in solution space section, a colour-coded polar plot for phase relationship between adjacent joint torques would enable us to identify postural control strategies. The possible coordination modes are shown around the circumference of Fig. 5. The Scatter of COP-COM relative phase data point in quadrant I or III represents an in-phase coordination pattern between the COP and COM motion and indicates that COP and COM move in the same direction anteriorly or posteriorly, respectively (Fig. 5). In contrast, the scatter of data point in quadrant II or IV shows anti-phase coordinate patterns meaning that COP and COM move in the opposite direction.

An increase in the polar plots’ radius represents progress in time or change in the frequency level. Therefore, during radius increment or decrement, data point transition from one colour zone to the other shows a transition from anti-phase to in-phase relationship or vice versa at an instant in time or frequency level. For example, the polar plots for phase relationship between adjacent torques show that this change from one colour to another represents a transition between available balance control strategies in terms of the ankle, hip, knee, or combined strategy, shown around the circumference of Figs. 6 and 7. The transition between light and dark versions of the same colour within the same phase coordination (i.e., in-phase or anti-phase) depicts a change from the first oscillator to the second oscillator dominancy, or vice-versa. For example, the polar plot for the COP-COM relative phase illustrates that the change from light to dark versions of the same colour represents a transition from COP dominancy to COM dominancy as shown around the circumference of Fig. 5. Collectively, investigation of phase relationships in a higher level of space via COP-COM relationship and lower level of synergies would help us depict transition in both posture stability and transition in balance control strategies, respectively.

**Figure 8:**
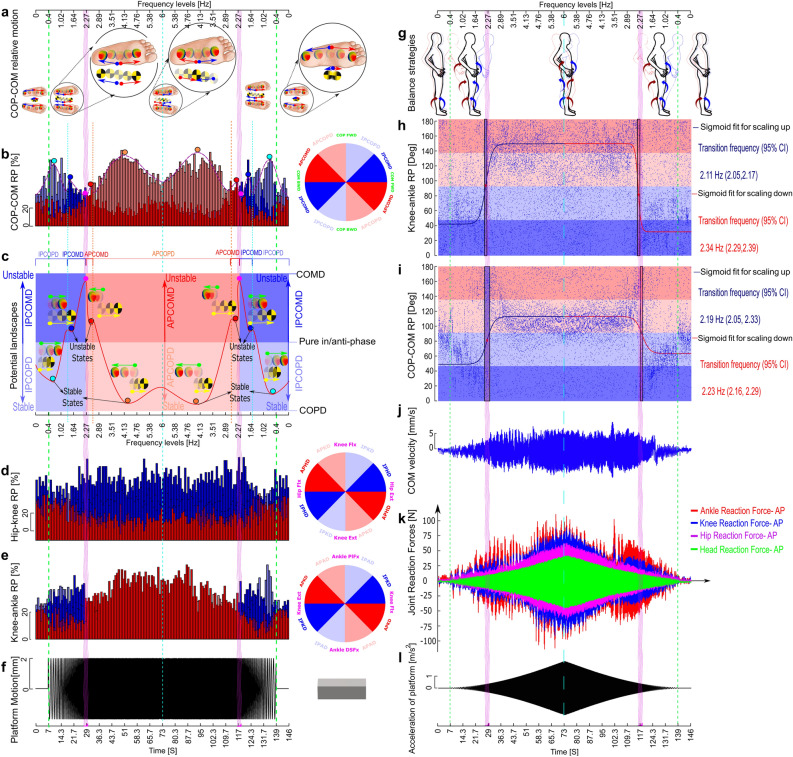
Transitions in coordination modes as a function of platform frequency. (**a**) Outcome coordination modes for COP-COM relative motion which are signified by red and blue arrows for FW and BW motion of body, respectively. (**b**,**d**,**e**) Relative phase rate (RPR) graphs as a function of the platform frequency for the COP-COM, hip-knee torque and knee-ankle torque relative phase, respectively. The RPR graph is a three-dimensional bar chart where the x-axis signifies the frequency level, the y-axis corresponds to data for one of the four colour-coded coordination patterns shown on the right-hand circles, and the z-axis shows the percentage of each coordination pattern used in that specific window or frequency level. In other words, the percentage of each coordination pattern at each window is assigned to a colour based on the polar position within the coloured coordination pattern classes. (**c**) Potential landscapes of the stable and unstable pattern dynamics^9^. The red and blue colours signify anti-phase and in-phase relationship between COP and COM, respectively. Blue and red balls show unstable states, while cyan and orange balls show stable states. The pink balls represent the absolute maximum and transition points in two frequency increasing and decreasing conditions. The cyan and orange dashed lines signify the transition between dominancy of COP and COM in anti-phase and in-phase mode, respectively, while the pink shaded area signifies the transition between anti-phase and in-phase mode. The green and yellow arrows signify relative motion of COP and COM in each stable and unstable well. The length of arrows signify the dominancy of COP/COM motion. (**f**) Platform displacement against frequency. (**g**) Transition between balance control strategies when platform frequency was scaled. (**h**,**i**) Sigmoid fits to colour-coded^26^ phase relationship data for knee-ankle joint torque and COP-COM motion. (**j**) COM velocity in AP direction. (**k**) head, hip, knee and ankle joint reaction forces. (**l**) Acceleration of platform against frequency.

Needham et al.^27^ introduced a coupling angle mapping which signified a colour-scale approach to display changes in coordination pattern classifications across a movement cycle. A similar colour-coded approach followed by a fitting method was used to capture the transition time visualized in polar plots precisely. This technique, called the relative phase rate (RPR) graph, involved taking consecutive subsets or windows of data with the length of 100 samples (1 s) from the relative phase data and calculating the percentage of each of the four colour-coded coordination patterns over these windows. Then, a three-dimensional bar chart where the x-axis shows the number of windows, the y-axis corresponds to data for one of the four colour-coded coordination patterns, and the z-axis shows the percentage of each coordination pattern used in each specific window (Fig. 8b–d,e). In other words, the percentage of each coordination pattern at each window was assigned to a colour based on the polar position within the coloured coordination pattern classes. A nonlinear fit of a sigmoid function to the relative phase data was later used to find the transition time between available strategies or coordination modes precisely^43^. The transition window identified via the windowing method, the RPR graph, was later used to set the starting point or initial values for the sigmoid fitting method to estimate the sigmoid parameters, including the transition point more optimally.

### Computing joint reaction forces and net torques

3D inverse dynamic analysis (IDA) was performed using a Newton-Euler method^40^. Body segment inertial properties, kinematics, and external forces were substituted into the equation of motions to determine the joint reaction force at centre of rotation of the ankle, knee, hip and at the head; and the net joint torque about the ankle, knee and hip joint^40^.

### Potential landscape of the stable and unstable dynamics

To develop an intuitive understanding of the COP-COM dynamic behaviour, we propose a potential landscape of the stable and unstable dynamics that is similar to those proposed in Kelso et al. study ^9^. Simply at this point, we used the dashed curve in Fig.  8b for COP-COM relative motion and mirrored it to create potential landscapes of the stable and unstable pattern dynamics as shown in Fig. 8c. The balls in Fig. 8c, identify the state of the system and the wells of the landscape identify basins of attraction. Depths of the potential wells in Fig. 8c , could indicate the strength (or stability) of attractor in the given coordination mode. Higher variability in relative phase modes contribution would be signified by a peak in the landscape.

### Statistical analysis

The normality of data was checked using the Kolmogorov-Smirnov test. A 2-way repeated-measures ANOVA of *2 (direction of platform frequency change: increasing/decreasing) * 10 frequency levels (0, 0.4, 1.02, 1.64, 2.27, 2.89, 3.51, 4.13, 4.76, 5.38, 6 Hz) was conducted on the COP-COM and knee-ankle relative phase data to investigate significant difference in phase relationship across frequency levels. The two-way ANOVA was used to analyse the influence of platform frequency and scaling direction on knee-ankle torque and COP-COM relative phase. Then, the Bonferroni post hoc test was employed to determine the differences in all paired comparisons for the dependent variables. A paired t-test also was used for the transition frequencies indicated by the knee-ankle torque and COP-COM phase relationship signals in increasing and decreasing conditions to investigate hysteresis effects for the two platform conditions. For both the t-test and ANOVA, the p-value of 0.05 was chosen as a significance level.

## Results

### Polar plots

The polar plot in Fig. 5 illustrates that as the platform frequency increases, the COP-COM relative phase moves from in-phase to anti-phase relationship. This can be observed by tracking the change in position of data points in different coloured zones while we move along the radius from the centre to the halfway point at 6 Hz. In quiet standing (radius is between 0 (centre) and 0.4 Hz level), COP and COM have an in-phase relationship with COP dominancy (IPCOPD, around the circumference of Fig. 5) as the green-colour data points are scattered in light blue portions. When the platform oscillates in the frequency range between 0.4 and 2.27 Hz, COP gradually loses its dominancy to the COM as the COP-COM moves from IPCOPD relationship to an in-phase relationship with COM dominancy (IPCOMD) (the blue-colour data points are gradually scattered from light to dark blue portions in this frequency range). When platform frequency reaches critical frequency levels between 2.27 and 2.89 Hz, the data points are scattered in dark red portions. This shows that COP-COM switches from IPCOMD relationship to an anti-phase with COM dominancy relationship (APCOMD). By increasing platform frequency from 2.89 to 6 Hz, COP gradually regains its dominancy through an anti-phase relationship with COM (APCOPD) as the red-colour data points are scattered in light red portions. From the point where the platform frequency is scaled down from 6 Hz, the COP keeps its dominancy (APCOPD relationship) until the frequency level of 2.89–2.27 Hz is reached. COM gradually becomes dominant at this frequency range (APCOMD relationship). At the critical frequency range between 2.27 and 1.64 Hz, the COP-COM transits back to the in-phase relationship with COM dominancy (IPCOMD relationship). Finally, when the platform frequency reaches the frequency level below 1.02 Hz, COP becomes dominant again and the COP-COM returns to IPCOPD relationship.

Figure 6 represents the polar plot for phase relationship between hip and knee joint torques for a representative participant. While the frequency of the platform scaled up and down between 0.4–6 and 6–0.4 Hz, the hip and knee coordination generally remains in-phase (coded by the high distribution of VC angle data points in blue portions). As illustrated in Fig. 4c, the in-phase relationship between hip and knee joint torques identifies that either ankle or knee strategy has been attempted by the participant depending on the phase relationship between knee-ankle joint torques.

Figure 7 shows that knee-ankle joint torques move from in-phase relationship (blue portions) to anti-phase relationship (red portions) in the frequency range between 2.27 and 2.89 Hz during scaling frequency up from 0.4 to 6 Hz. Considering the possible balance control strategies shown around the circumference of the polar plot, and the in-phase hip-knee relationship, this transition from in-phase to anti-phase relationship signifies a transition from ankle to knee strategy^19^ at this specific frequency range. During scaling frequency back down from 6 to 0.4 Hz, the knee-ankle joint torques transit back from anti-phase relationship (red portions) to in-phase relationship (blue portions), in the frequency range between 2.27 and 1.64 Hz, which represents a transition from knee back to ankle strategy. This pattern was present across all participants, even though individual-specific transition frequency was observed (mean and 95 percent confidence interval (CI) of the transition frequencies for participants’ mean coordination data are shown by vertical red and blue bars in Fig. 8h–i). The transition frequency of individual participants for COP-COM and knee-ankle phase relationship is shown in Supplementary Table 1 online. 66% of the participants showed a transition from in-phase to anti-phase COP-COM relationship after a transition from ankle to knee strategy in frequency increasing condition, while 89% of the participants transited from anti-phase to in-phase COP-COM relationship after a transition from knee to ankle strategy in decreasing condition.

### Relative phase rate (RPR) graphs and sigmoid fits

To quantify the transition frequency region depicted in the polar plots, we implemented the relative phase rate (RPR) method on the relative phase data point for each participant (Fig. 8b, d–e). A transition from blue bars to red bars signifies a transition from in-phase to anti-phase relationship and vice versa. When scaling frequency up from 0.4 to 6 Hz, the transition frequency from in-phase to anti-phase relationship was found in a frequency window of 2.19 Hz and 2.27 Hz for knee-ankle and COP-COM phase relationships, respectively. When scaling frequency down, the transition from anti-phase to in-phase relationship occurred in frequency windows of 2.27 Hz and 2.19 Hz for knee-ankle and COP-COM relative phase relationships, respectively. To identify transition frequency precisely, we used the transition windows to set the starting points or initial values for the fitting method (Fig. 8h–i), to estimate the sigmoid parameters, including transition point (frequency), more optimally. Results show that, during scaling frequency up, the transition frequency from in-phase to anti-phase relationship was found at a frequency of 2.11 Hz and 2.19 Hz for knee-ankle and COP-COM phase relationships, respectively. During scaling frequency down, the transition frequency from anti-phase to in-phase relationship was found at 2.34 Hz and 2.23 Hz for knee-ankle and COP-COM phase relationships, respectively. In order to implement the sigmoid fit method, each relative phase data point was translated from ${{0}^{\circ }}\le \theta \le {{360}^{\circ }}$ to ${{0}^{\circ }}\le \theta \le {{180}^{\circ }}$. There are two sigmoid fits for each relative phase data, one for scaling frequency up and one for scaling down condition. The sigmoid fits identified the exact transition frequency for each relative phase data. Figure 8g represent the transition between balance control strategies under the effect of scaling platform in a chirp pattern shown in Fig. 8f.

As discussed in the “Methods” section, deeper wells in a potential landscape indicate more stable modes. Therefore, the potential landscape presented and discussed in Fig. 8c demonstrates four coordination modes or states, two stable states and two unstable states (pointed out by black arrows), for each direction of platform frequency change. As shown in the landscapes, in stable states (cyan and orange balls, $\sim$ 0.6 and 4. 20 Hz, respectively), COP dominates, while in unstable states ( blue and red balls, $\sim$ 1.5 and 2. 5 Hz, respectively), COM dominates.

### The joint reaction forces and COM velocity

As the platform frequency is scaled up from zero to a transition frequency range signified by pink shaded areas, the platform’s acceleration increases (since acceleration goes with square of frequency for constant displacement amplitude) and induces a similar growth in all the joint reaction forces (Fig. 8k). The hip and head joint reaction forces continue to grow and drop similarly with acceleration even after the transition region. However, it appears that the ankle and knee joint reaction force and COM velocity flatten off once we reach the transition frequency window (Fig. 8j–k). Since then, the knee and ankle joint reaction force and COM velocity fall off until they reach a frequency level of 3.51 Hz where they return to the increasing pattern again up to a frequency of 6 Hz. When the platform frequency is scaled back down from 6 to 0, the ankle and knee, joint reaction forces, and COM velocity represent a similar behaviour as shown in Fig. 8.

There is a significant main effect of frequency for knee-ankle relative phase rate, F(9,160) = 10.99 at $p=0.023$. No significant main effects of the change direction are observed on the knee-ankle relative phase, F (1,160 ) = 0.48. The interaction of frequency and direction of change was found not to be significant, F(9,160) = 0.46. The Bonferroni post hoc test on the knee-ankle relative phase showed that comparisons between the first three frequency levels with the last six frequency levels are significant at the 0.05 level for platform frequency. Similarly, the two-way ANOVA test on COP-COM relative phase data showed a significant main effect of frequency F(9,160) = 3.3 at $p=0.016$. The main effect of direction of change on the COP-COM is not significant, with F(1,160 ) = 2.59 at $p=0.10$. The interaction of frequency and direction of change is not significant, F(9,160) = 0.53. The Bonferroni post hoc test on the COP-COM RP rate showed that the interaction is due to comparisons between the first four frequency levels, with the last five frequency levels being significant at the 0.05 level for platform frequency. A paired sample t-test at the transition frequency of each platform condition showed that the relative phase of COP-COM and knee-ankle relationship differs significantly ($p= 0.031$) for both increasing and decreasing platform frequency conditions.

## Discussion

Our findings revealed two balance control strategies: an ankle and a knee strategy. These strategies were employed sequentially, with a transition frequency at around 2.11 and 2.34 Hz during scaling up and down, respectively. The transition between knee-ankle strategies was followed by a transition from in-phase to anti-phase relationship for the COP-COM motion at specific frequencies (2.19 and 2.23 Hz during scaling up and down, respectively), also demonstrating hysteresis in the transition frequency.

### The knee balance control strategy

The ankle strategy, characterised by in-phase relationship between knee-ankle and hip-knee torque, prevailed at frequencies below 2.11 Hz and 2.34 Hz (in increasing and decreasing conditions, respectively). The knee strategy, characterised by anti-phase knee-ankle and in-phase hip-knee torque, was employed at frequencies above 2.11 Hz and 2.27 Hz (in increasing and decreasing conditions, respectively).

To our knowledge, this is the first study that has identified knee strategy in maintaining postural control in perturbed balance tasks. Previous work has suggested that a hip strategy would follow the ankle strategy^3,16,44,45^. We suggest that our novel finding is likely due to (1) the smaller amplitude of floor motion used compared to previous studies, (2) methodologies used in previous studies. In study described here, acceleration of platform was scaled from 0 to 0.25 *g* as the platform accelerations $>0.25~g$ are rarely seen in civil structures in operation. However, when the large displacements used by previous studies, ranging from 9 to 23 cm, are translated to acceleration, they are rarely representative of any civil structures. Ko et al. ^10^ perturbed participants in AP direction at floor amplitude of 10 cm, 16 cm and 23 cm and observed a transition from ankle to ankle-hip mode (similar to a hip strategy) at frequencies higher than 0.94 Hz in the 16 and 23 cm conditions. Similarly, Mohd et al.^13^ used platform amplitude of 10 cm in AP direction and showed a transition from ankle to hip strategy at frequencies higher than 0.4–0.6 Hz. Finally, Runge et al.^16^ showed a transition from ankle to hip strategy at velocities $>20$ cm/s. Iqbal et al.^46^ showed that the hip strategy might only have a greater advantage when the COM is located at the forefoot ,^3,5,45,47–49^or during larger perturbations, when the COM might be displaced near the base of support (BOS) boundaries and requires larger adjustment in COM motion through the hip strategy^16^. However, relatively small floor movement of 4 mm in this study which is in the range of real-world situations, might not place the COM outside the BOS or into the forefoot. Therefore, the balance system may not require extensive changes in the COM AP position using a hip strategy. In this study, the knee strategy appears to be employed when participants need to move COM slightly and quickly in the AP direction in response to the platform’s small (4 mm) and fast motion (higher than 2.11–2.27 Hz). This can be seen in Fig. 8j, where the growing speed of COM motion in the AP direction has been arrested using a knee strategy at frequencies higher than 2.11–2.34 Hz. In addition, the knee strategy may provide another advantage over the hip strategy when underfloor motion is of smaller amplitudes as it may result in a smaller head movement than the hip strategy and may require smaller eye movements to compensate. In this way, the vestibular and visual systems may receive better sensory feedback ^50^.

Secondly, previous studies assumed that the knee joints make a negligible contribution to the recovery of balance by the postulated control mechanisms^3^. This may be an oversight, since we have attempted to provide the 3D solution space (Fig. 3), created by the interactions between joint torques of the lower limbs which illustrated that ankle, knee, hip, and combined strategies are possible solutions to balance control offered by CNS that could emerge under different environmental conditions^41^.

In line with discussion point of Ko et al. ^6^, a formal biomechanical explanation of balance control strategies is required. Ko et al. suggested that stiffening the ankle joint is sufficient to maintain the COM in the equilibrium position at low translation frequencies, but at high translation frequencies compensatory movement at the ankle joint alone is not sufficient to dissipate the increased inertial force. Thus, participants recruited additional DOF such as hip and knee joint motions to dissipate the increasing forces induced by platform translation and prevent transmission of the platform motion to the upper body. On the contrary to Ko et al.^6^ study, our findings show that platform acceleration is transmitted to upper body (head and hip joints) linearly, Fig. 8k–l at all frequency levels. However, a nonlinear transmission of the acceleration to the ankle and knee joint is observed. In other words, in the ankle strategy region, where the body moves as an inverted pendulum, all the joint reaction forces grow proportionally with a similar pattern with acceleration. This has induced an in-phase relationship between COP-COM (Fig. 8a). However, as we reached the transition region, maintaining balance in the ankle joints tends to become insufficient^44^. Thus, participants change their strategy to a knee strategy, resulting in an immediate decrease in the ankle and knee joint reaction forces (Fig. 8k). Finally, this transition from ankle to knee strategy has produced an anti-phase relationship between COP and COM motion (Fig. 8a) and arresting COM growing speed (Fig. 8j).

### Regulation of control strategies by COP-COM relative motion

Our findings revealed that COP-COM moves from an in-phase to an anti-phase relationship at frequencies above 2.19–2.23 Hz. This result was consistent with previous studies^10,11,13^ that showed the same transition at frequencies ranging from 0.4–0.6 Hz. However, it was not clear from previous studies why COP-COM transitions from in-phase to anti-phase relationship under the effect of increasing frequency or vice versa. We postulated that when COM starts to dominate the COP, the system becomes mechanically unstable and is likely a mechanical reason for the change in strategy, since a common assumption is that COP is the controlling variable, and COM is the controlled variable^32,33^. Indeed, our findings revealed via VC that when the COM takes dominancy over COP it causes a change in the balance control strategy. As such, COP-COM coordination exhibited four modes sequentially: in-phase with COP dominancy, in-phase with COM dominancy, anti-phase with COP dominancy, and anti-phase with COM dominancy. Specifically, Fig. 8b shows that initially, at lower frequencies, COP and COM move in-phase while the COP motion is dominant (IPCOPD, light blue bars); however, once platform frequency reaches 1.32 Hz, signified by cyan dashed line, COP loses its dominancy to COM (IPCOMD, dark blue bars). COM keeps its dominant in-phase relationship with COP until platform frequency reaches 2.19 Hz, where COP represents an anti-phase motion with COM dominancy (APCOMD, dark red bars) which likely represents a loss of stability in the balance control system. The instability induces a new search process for a new stable region and leads the postural system to generate an adaptive compensatory coordinative mode at the joint level. By further incrementing platform frequency, COP regains its dominancy through the anti-phase relationship with the COM (APCOPD, light red bars) at a frequency level of 2.53 Hz, signified by the red dashed line, indicating a stable solution of the system until the maximum platform frequency of 6Hz is reached. This scenario is reversed when the platform frequency decreased with the transition happening at a slightly higher frequency of 2.23 Hz, demonstrating evidence of hysteresis.Mapping this to the joint level, following the ankle strategy, participants bend their knees and move their body similar to a double inverted pendulum through a knee strategy. The knee strategy displaces the COM slightly in the AP direction; however, it induces the anti-phase motion between COP and COM. By doing so, the fast movement of COM is slowed down and finally lets the COP displacement take over again, resulting in a more stable balance control strategy. The synergies of the musculoarticular system at lower-level generate higher-order variables such as the COP and COM interaction. Our results show that the COP-COM transitions followed the transition between knee-ankle joint torques. This work, alongside with Ko et al.^10^, provides evidence that the COP-COM motion is the global variable for human balance control system as the transition in COP-COM coordination occurred after the underpinning variables such as coordination between joint angular motions.

### Intuitive understanding of the COP-COM dynamic behaviour

The potential landscape for COP-COM dynamic behaviour, Fig. 8c, showed that the wells for COP dominancy (cyan and orange balls, pointed out as stable states) were much deeper than those for COM dominancy mode (red and blue balls, pointed out as unstable states). This could indicate more stable coordination between COP-COM when COP is dominant. When comparing stable states (orange and cyan balls), the anti-phase mode (orange balls, $\sim$ 4.20 Hz) had deeper wells than the in-phase mode (cyan balls, $\sim$ 0.6 Hz). This could indicate stronger attractor for anti-phase COP-COM relationship with COP dominancy, requiring greater change in the control parameter (frequency) to change the layout or the stability of the attractor. Our finding showed that, the transition from in-phase to anti-phase COP-COM relationship corresponded to the transition from ankle strategy to knee strategy. Therefore, the weaker in-phase coordination for COP-COM might indicate that the ankle strategy provides larger instability or weaker coordination. The proposed potential landscape moves us towards the model that will inform our predictions of changes in strategy with changes in environmental conditions, as well as providing evidence for the theoretical perspective of nonlinear phase transitions in human movement. Future analysis might be able to derive a potential function governing the dynamics of the balance system and then create the potential landscape quantitatively.

## Conclusion

Our findings reveal three important features of dynamic postural control: (a) knee strategy is required to deal with the small-amplitude underfloor movement. Based on our current findings, the assumption that the body behaves with ankle strategy when the floor motion is small might need to be revised when frequency of motion exceeds 2.1–2.4 Hz. (b) The changes in dominancy of COP/COM motion can be representative of a change in the stability of the balance control system. We postulate that when COM starts to dominate the COP, the system is mechanically unstable. In this situation, another balance control strategy at the level of musculoarticular system (or joint torques) is required. Thus, participants bent their knees through the knee strategy which led to an anti-phase motion between the COP and COM and helped the COP to recover its dominance over the COM. This may provide new insight into future balance control criteria and modelling. The CNS goal might not be only to keep COM within BOS but rather to maintain the dominance of the COP motion. (c) Using the dynamical systems theory perspective of linearly scaling platform frequency and continuously monitoring the change in balance control strategy, a nonequilibrium phase transition from in-phase to anti-phase behaviour was observed for both COP-COM relative motion and knee-ankle torque. Our results present four new modes in the balance control system, including in-phase and anti-phase, with dominancy of one of the oscillators at both higher levels of space and lower levels of synergies.

## Future work

Future work should continue to explore the landscape of postural coordination modes through examining postural control strategies across a wide range of platform amplitudes and frequencies. We propose to employ 2D bifurcation analysis to model this landscape and shed light on postural strategies and possible transition frequencies or amplitudes. For the purposes of comfort and safety for a number of different populations, the design of structures such as buildings, transport, bridges, etc. should consider if frequency and amplitudes of floor motion elicit unstable and higher DOF balance control strategies.

## Supplementary Information

Supplementary Information.

## Data Availability

The datasets generated during and/or analysed during the current study are available in the University of Exeter Repository (ORE). For any other data query, please get in touch with the corresponding author.
