# Supplementary material for: Vector coding reveals the underlying balance control strategies used by humans during translational perturbation
Source: Sci Rep. 2022 Dec 5;12:21030. doi: 10.1038/s41598-022-24731-3 (PMC9722668; doi:10.1038/s41598-022-24731-3)
Supplement: Supplementary file 1 — Supplementary Information. [file 41598_2022_24731_MOESM1_ESM.docx]

**Vector coding reveals the underlying balance**

**control strategies used by humans during**

**translational perturbation**

Naser Taleshi, James MW Brownjohn, Sarah E Lamb, Stana Zivanovic, and Genevieve

KR Williams

Table 1 Participant specific transition frequency for the COP–COM coordination and knee-ankle joint torque coordination in the increasing and decreasing platform frequency conditions.

| Participants | Transition Frequency (Hz) | | | |
| --- | --- | --- | --- | --- |
|  | COP–COM | | Knee- Ankle | |
|  | Increasing | Decreasing | Increasing | Decreasing |
| 1 | 2.16 | 2.27 | 2.10 | 2.34 |
| 2 | 2.28 | 2.34 | 2.20 | 2.41 |
| 3 | 2.28 | 2.29 | 1.97 | 2.64 |
| 4 | 2.36 | 2.37 | 2.19 | 2.38 |
| 5 | 2.18 | 2.32 | 2.20 | 2.62 |
| 6 | 2.12 | 2.19 | 2.28 | 2.70 |
| 7 | 1.95 | 2.08 | 2.29 | 2.20 |
| 8 | 2.50 | 2.13 | 2.28 | 2.30 |
| 9 | 2.50 | 2.44 | 2.08 | 2.21 |
